# Artificial intelligence integrates multi-omics data for precision stratification and drug resistance prediction in breast cancer

**DOI:** 10.3389/fonc.2025.1612474

**Published:** 2025-09-12

**Authors:** Deshui Ran, Jing Li, Mengmeng Zhao, Li Du, Yang Zhang, Jida Zhu

**Affiliations:** ^1^ Department of Imaging, Jinan Second People’s Hospital, Jinan, China; ^2^ Department of Acupuncture and Moxibustion, Shandong College of Traditional Chinese Medicine, Yantai, China; ^3^ Breast and Thyroid Surgery, Shandong University of Traditional Chinese Medicine Affiliated Hospital, Jinan, China

**Keywords:** breast cancer, artificial intelligence, multi-omics, immunotherapy, precision stratification, drug resistance

## Abstract

Breast cancer (BC), the most prevalent malignancy in the female population, often presents significant difficulties in early diagnosis and identification of molecular subtypes. In addition, due to the lack of obvious clinical symptoms in the early stage and the lack of effective early detection means or specific biomarkers, about 30% of the cases are already in the advanced stage at the time of diagnosis, which directly leads to the patients missing the best treatment period. Unfortunately, BC is also highly heterogeneous, and its different molecular typing directly affects the outcome of treatment regimens such as chemotherapy, immunotherapy, etc., and significantly correlates with patients’ 5-year survival rates. Artificial intelligence (AI) has rapidly advanced from proof of concept to prospective and real-world deployments, delivering radiologist level accuracy, improved specificity, and substantial workload reduction (≈44%–68%) without compromising cancer detection. Some studies even report more cancers detected when AI supports readers. These gains translate into earlier diagnosis, fewer unnecessary recalls, and more efficient screening workflows. Concurrently, multi-modal AI (integrating mammography, ultrasound/DBT, MRI, digital pathology, and multi omics) enables robust subtype identification, immune tumor microenvironment quantification, and prediction of immunotherapy response and drug resistance, thereby supporting individualized treatment design and drug discovery. The aim of this review is to systematically illustrate the efficient application of AI technology in BC diagnosis, such as constructing early diagnostic models based on multi-omics data, identifying molecular subtypes of BC, quantifying the tumor immune microenvironment and predicting the immunotherapeutic response, as well as investigating drug resistance of BC and developing new therapeutic agents. In the future, AI technology will be able to provide more accurate individualized diagnosis and treatment for BC patients.

## 1 Introduction

BC, as one of the most common malignant tumors worldwide, is also the second leading cause of cancer-related deaths worldwide (1). By 2022, there will be more than 600,000 BC-related deaths globally, mainly in developing countries (2, 3). Unfortunately, the incidence of BC continues to rise, as does the mortality rate due to the lack of effective early detection techniques. Clinical retrospective studies have found that the 5-year survival rate for BC patients diagnosed at an early stage is 90%, whereas once a patient reaches an advanced stage, their survival and quality of life decreases significantly (4, 5). Clinically, traditional screening techniques such as digital mammography still have numerous limitations, and many elderly women have decreased tumor detection rates due to high breast density (6). BC exhibits pronounced molecular and immune heterogeneity (spanning ER/PR/HER2 status, PAM50 classes, proliferative activity, and TIME), which drives variable response to chemotherapy, targeted agents, and immunotherapy. Conventional imaging alone struggles to capture this heterogeneity, and performance is further hindered in women with dense breasts, where lesion conspicuity decreases and recall may increase (7, 8). Therefore, the integration of current multi-omics data as well as the development of new diagnostic, and prognostic technologies are crucial for the comprehensive diagnosis and treatment of BC.

AI is currently experiencing rapid growth, especially machine learning (ML), has provided new and important directions for precision diagnosis and treatment of BC (9). By extracting quantitative features from medical images and combining them with ML algorithms, imaging histology can reveal tumor heterogeneity in a finer way. It provides an important reference for clinical auxiliary diagnosis, prognosis evaluation and treatment decision (10, 11). In the field of BC radiomics, AI has been successfully applied to a variety of imaging modalities, including Ultrasound, X-ray, MRI etc. By analyzing the dual-energy images of contrast-enhanced mammography (CEM), ML algorithms can improve the detection rate of tiny lesions and significantly reduce problems such as false positives (12, 13). In addition, the introduction of ML further optimizes its ability to identify tumor angiogenesis in dense breasts. Interestingly, with the rapid development of deep learning (DL), more and more multimodal data are continuously being integrated (14). This integration of multimodal data can further enhance the accurate prediction of BC diagnosis, immunotherapy response, molecular typing and prognosis by ML models. By extracting high−throughput radiomic features or learning deep representations directly from images, DL can enhance benign–malignant discrimination and reduce unnecessary biopsies. Act as a non−invasive surrogate for molecular subtypes and actionable mutations. Predict axillary lymph−node (ALN) status and metastatic burden and estimate therapeutic response, including pathological complete response (pCR), immune phenotypes (e.g., PD−L1, TILs), and risk of relapse. Beyond single−modality modeling, multi-modal and longitudinal integration—combining mammography, ultrasound, and multiparametric MRI with PET/CT, digital pathology whole−slide images, and multi−omics (bulk RNA−seq, single−cell profiles)—has emerged as a key strategy to link imaging phenotypes with tumor biology and the TIME, thereby supporting individualized decision−making (15).

In this review, we summarize the role of ML in BC radiomics and imaging histology in detail. The application of ML in BC diagnosis, molecular typing, prognosis and response to immunotherapy is summarized in focus. In the future, by integrating multi-omics data, ML will provide new ideas and important theoretical references for precision treatment of BC patients.

## 2 AI-driven workflow for BC

### 2.1 Significance and clinical motivation

Computational radiomics transforms routine breast imaging into reproducible, quantitative biomarkers that support earlier detection, biologically informed risk stratification, and more efficient screening workflows. Recent prospective and real-world studies show that AI-assisted reading can maintain or improve cancer detection while substantially reducing screen-reading workload often by ~45–50%, which directly addresses workforce constraints and helps shift diagnoses to earlier, more curable stages. These benefits motivate an end-to-end, standards-aligned workflow to ensure reliability, comparability, and safe clinical translation (16, 17).

### 2.2 Data quality and standardization

High-quality, standardized imaging is foundational. Multi-modal acquisitions (mammography (MG), ultrasound (US/ABUS), multiparametric MRI, and metabolism-sensitive PET/CT) capture complementary morphologic, functional, and metabolic information. Protocol harmonization should follow community guidance from the Quantitative Imaging Biomarker Alliance (QIBA) and the Quantitative Imaging Network (QIN); downstream feature extraction should adhere to the Imaging Biomarker Standardization Initiative (IBSI), which provides consensus definitions, benchmark datasets, and reference values for 169 features to enable cross-software verification (18, 19). Preprocessing typically includes intensity normalization, noise/artifact mitigation, inter-modal or longitudinal registration, and resampling to a common voxel grid, with transparent reporting of all parameters.

### 2.3 Segmentation and ROI strategy

Accurate delineation of the lesion and relevant peritumoral tissue determines downstream feature quality. Manual or expert-edited semi-automatic approaches remain strong references but are labor-intensive and subject to inter-reader variability; fully automated deep segmentation (e.g., U-Net variants) offers scalability but must be stress-tested under domain shift. Beyond whole-tumor masks, habitat and peritumoral strategies (e.g., core vs rim; fixed-width peritumoral rings) capture microenvironmental heterogeneity and have shown added value for subtype differentiation, nodal status prediction, treatment response, and prognosis. Robustness should be confirmed with external, multi-center cohorts (20, 21).

### 2.4 Feature engineering and deep representations

From segmented ROIs, radiomics derives shape, first-order histogram, and higher-order texture features (e.g., GLCM/GLRLM/GLSZM/NGTDM), as well as transform-domain descriptors (e.g., wavelet, LoG). Open-source PyRadiomics (IBSI-compliant) is widely adopted and facilitates transparent, reproducible extraction and reporting. In parallel, deep learning (CNNs, Vision Transformers) learns hierarchical image representations directly from pixels. In breast cancer, hybrid models that combine engineered radiomics, deep embeddings, and clinical variables frequently improve robustness, interpretability, and generalization across vendors and centers (22).

### 2.5 Feature selection and model construction

Because engineered and learned features are high-dimensional and partially redundant, rigorous feature reduction is essential. Common practice includes univariate filtering and correlation pruning, stability checks under resampling/discretization choices, embedded sparsity methods, and tree-based importance ranking. Classifiers/regressors typically include LR, SVM, RF, gradient boosting (e.g., XGBoost), and deep networks; survival tasks frequently use Cox-based or deep survival models (23, 24). Model choice should balance discrimination, calibration, interpretability, and computational efficiency, with case-level explanations (e.g., feature attribution or saliency) to support clinical acceptance.

### 2.6 Validation, calibration, and clinical utility

Generalizability must be demonstrated through nested cross-validation, temporal splits, and external multi-center testing, ideally with harmonized protocols. Beyond discrimination (e.g., AUC), best practice includes calibration assessment (reliability curves, Brier score), reporting of workload and recall metrics in screening settings, and decision-analytic evaluation (decision curve analysis) to show net clinical benefit over “treat-all/none” across plausible thresholds. These elements connect algorithmic scores to patient-relevant actions, resource use, and health-system outcomes (25).

### 2.7 Translational considerations: generalizability, fairness, and privacy

**Figure 1:**
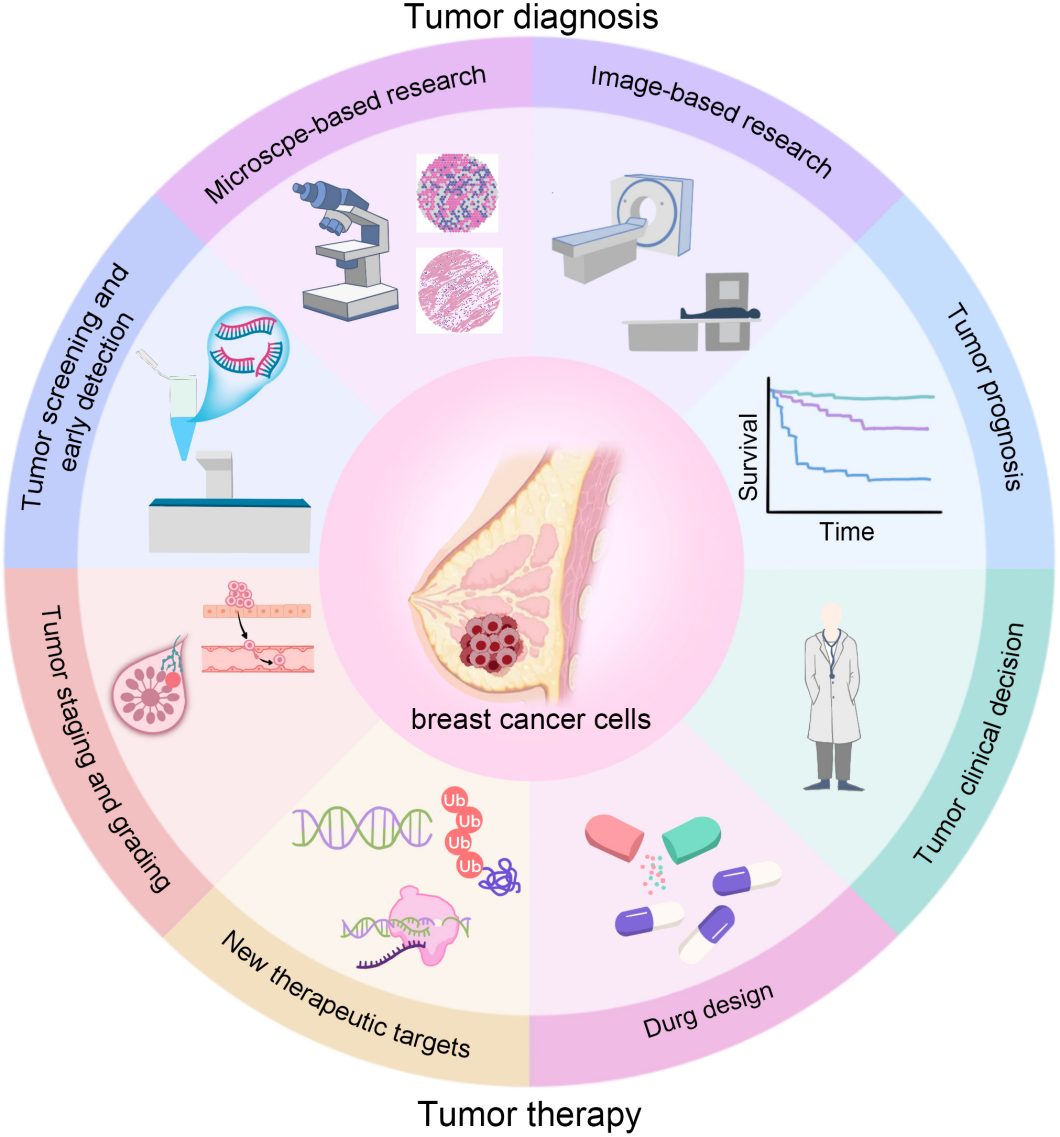
The workflow of AI in BC.

Successful deployment requires continuous monitoring of drift, subgroup performance, and fairness across scanners, sites, and demographics. Federated learning (FL) and privacy-preserving analytics enable multi-institutional model training when data sharing is constrained and have shown feasibility in breast imaging, approaching centralized performance while mitigating transfer risks. Large national programs and trials are underway to evaluate accuracy, workload, safety, and equity at scale, underscoring the need for prospective designs, governance, and transparent reporting (26, 27). Only through rigorous model validation can we ensure that the constructed imaging genomics models have sufficient robustness, reliability and generalization ability, and lay a solid foundation for the final clinical translation and application. Here, we summarize the workflow of AI in breast cancer radiomics (**Figure 1**).

The diagram summarizes how AI supports the care pathway: screening and early detection (higher or maintained cancer detection with lower reading workload); diagnosis (lesion segmentation and benign–malignant classification, including dense breasts); staging and prognostication (axillary status, tumor burden, and survival/recurrence risk); treatment decision-making (non-invasive molecular subtyping, prediction of pCR and immunotherapy response); and therapy development (prioritizing drug targets and assisting drug design). Multimodal data (mammography, ultrasound/ABUS, CEM, MRI, PET/CT, and pathology WSI) can be integrated to deliver calibrated, explainable outputs that link to concrete clinical actions (recall, biopsy, surgery, systemic therapy, and follow-up).

## 3 Precision diagnostic stratification: benign–malignant and molecular subtyping

### 3.1 Advances in AI for BC diagnosis

The joint use of DL with multimodal data such as imaging histology significantly improves the accuracy of benign-malignant differentiation of BC (28). Imaging histology, on the other hand, reveals tumor heterogeneity through high-throughput extraction of quantitative features from MG, MRI and ultrasound images. Research Findings. Using the combination of the DL model U-Net with case-based reasoning (CBR), the final model achieved an accuracy of 91.34% in the differential diagnosis of malignant masses by accurately segmenting mammography (MG) images (29, 30). In addition, a segmentation accuracy of 86.71% was simultaneously achieved, and its high-precision diagnostic performance and segmentation capability provide an important basis for interpretable clinical decision-making. In addition, a study by Gao and colleagues et al. proposed a new shallow-deep convolutional neural network (CNN) model that achieves 92% accuracy in classifying the benign and malignant nature of lumps in BC by phased processing of low-energy images with deep feature extraction (31). Further studies found that hybrid CNN architectures (e.g., ResNet50, AlexNet) combined with variance thresholding (TV) feature screening algorithms, through multi-class support vector machine (MSVM) classification, even achieved 99% accuracy in diagnosing BC on the mini-MIAS dataset (32). In addition, DL also achieved another high recall rate of 95.65% through cross-channel convolutional correlation features (33). The study showed that the gradient and gray scale covariance matrix (GLCM) features in the peri-tumor region could effectively differentiate benign and malignant lesions, and when combined with the normal tissue features of the contralateral breast, the classification AUC was improved from 0.79 to 0.84 (34). CEM-based imaging genomics model, with automated segmentation and three-compartmental quantification, achieved a positive predictive value of 0.49, which is 47% higher and 35.8% lower than that of conventional MG. 47% and reduced 35.8% of nonessential biopsies (35, 36). In multimodal MRI, the imaging histology model incorporating T2WI, ADC and dynamic contrast enhancement (DCE) sequences had a predictive efficacy of AUC 0.95 for HER-2 expression status, and after combining with the BI-RADS score, the AUC for BC diagnosis was elevated to 0.98, which provided a new method for non-invasive preoperative assessment (37). Chang et al. analyzed a single-reader national screening setting in Korea, enrolling 24,543 women, with 140 cancers detected (0.57%) over one year. For breast-imaging subspecialists, AI-CAD increased the cancer detection rate (CDR) by 13.8% (5.70‰ vs 5.01‰; p<0.001) without a significant change in recall rate (RR) (4.53% vs 4.48%; p=0.564). AI-CAD additionally detected 6 DCIS and 11 invasive cancers, and significantly improved detection of tumors <20 mm (p=0.002), node-negative disease (p=0.001), Luminal A subtype (p=0.002), and low-grade IDC (p=0.009). In simulated analyses for general radiologists, CDR increased by 26.4%, accompanied by a higher RR (6.89% vs 6.31%, p<0.001). Stand-alone AI achieved a CDR of 5.21‰ but with a higher RR (6.25%) (38). Qian et al. developed a multimodal risk-stratification model using clinical data + mammography (MG) + tri-modal ultrasound, trained on a multicenter dataset of 5,025 patients, 5,216 breasts, and 19,360 images, and prospectively validated in 187 patients (191 breasts). The model performed comparably to experienced radiologists for benign–malignant classification and outperformed them for finer pathological stratification. In the prospective cohort, the overall accuracy was 90.1%, close to the 92.7% accuracy of pre-biopsy pathological assessment, demonstrating real-world usability and transferability (39). Despite the outstanding performance of AI technology in improving diagnostic accuracy and multimodal data fusion, its clinical application still faces challenges such as insufficient standardization, limited interpretability and lack of large-scale validation. As summarized in **Table 1**, applications of AI in early detection and diagnosis are overviewed.

**Table 1:** The application of AI in early detection and diagnosis of BC.

Study & cohort	Model/method	Key results	Ref.
U-Net + CBR; MG images	Segmentation + case-based reasoning	Malignancy classification Acc 91.34%; segmentation Acc 86.71%.	(28, 29)
Hybrid CNN features + variance thresholding (TV) + MSVM; mini-MIAS	ResNet50/AlexNet features + MSVM	Acc 99% on mini-MIAS (small curated dataset).	(31)
Cross-channel convolutional correlation features	Deep learning	Recall 95.65%.	(32)
Peritumoral GLCM/gradient + contralateral normal-breast features	Hand-crafted textures	AUC improved 0.79 → 0.84.	(33)
CEM radiomics with auto-segmentation and tri-compartment quantification	Radiomics model	PPV 0.49 (+47% vs MG); non-essential biopsies −35.8%.	(34, 35)
Multi-sequence MRI radiomics + BI-RADS	Radiomics + BI-RADS	AUC 0.95 for HER2; AUC 0.98 for diagnosis when combined with BI-RADS.	(36)
AI-STREAM (Korea; n=24,543)	AI-CAD assisting readers	For subspecialists, CDR + 13.8% (5.70‰ vs 5.01‰, *p*<0.001) with no RR increase (4.53% vs 4.48%); stand-alone AI CDR 5.21‰, RR 6.25%.	(37)
Multi-center 5,025 pts; prospective 187 pts)	Multimodal ML (BMU-Net framework)	Prospective Accuracy 90.1%; comparable to experts for benign–malignant, superior for fine-grained pathology; close to pre-biopsy pathology 92.7%.	(38)

### 3.2 AI in molecular subtypes of BC

AI can significantly improve the highly accurate prediction of BC molecular staging by integrating multimodal imaging histology data. Deep models based on DCE-MRI imagingomics can achieve efficient prediction of BC molecular staging in a relatively short period of time by learning the features of intratumoral reinforcement texture and the time-signal intensity curve features (40, 41). Li and colleagues et al. showed that the DL model was capable of predicting ER+/-, PR+/-, HER2+/-, and triple-negative subtypes of BC by integrating MRI image data. The AUCs of the DL model for ER+/-, PR+/-, HER2+/-, and triple-negative subtypes were 0.89, 0.69, 0.65, and 0.67, respectively, with a high correlation between intratumoral enhancement heterogeneity and ER status (42). Follow-up studies focused on further optimizing feature selection, and the DCE-MRI model classified Luminal B subtypes with an accuracy of more than 80%, and HER2-enriched BCs with a classification rate of 73% (43). CEM extracts tumor angiogenic features through iodine contrast enhancement and dual-energy imaging to achieve non-invasive prediction of molecular typing. A model constructed based on 15 CEM features in cephalocaudal (CC) view was 94% accurate in distinguishing Luminal from non-Luminal subtypes (44). Combining the imaging histological features of PET/CT with clinical indicators (e.g., Ki-67), the multimodal model predicted HER2 status and molecular typing with an AUC of 0.95, which was further improved to 0.98 after combining with the BI-RADS scores (45). It was found that the imaging features of peritumoral region (e.g., edge gradient, gray scale covariance matrix) could supplement the intratumoral information and enhance the discriminative ability of TNBC. Jiang et al. integrated 11 features (including 5 peri-tumor features) and constructed a TNBC classification model with an AUC of 0.72 in the external validation set, highlighting the importance of peri-tumor microenvironment analysis (46). In addition, PET/CT imaging histology combined with metabolic parameters (e.g., SUVmax) can predict the Ki-67 high expression status of TNBC and provide a basis for prognostic assessment. AI models reveal the biological basis of molecular typing by fusing imaging genomics and gene transcriptome data. For example, the combined model based on MRI and gene expression profiling predicts the PIK3CA mutation status (AUC 0.82) of Luminal-type BC, providing clues for targeted therapy (47). Meanwhile, the association analysis of multiparametric MRI features with immunohistochemical markers (e.g., PD-L1) has advanced clinical studies for immunotherapy response prediction (48). Despite the excellent performance of AI models in single-center validation (e.g., 94% accuracy of CEM model), the generalization ability of multicenter data is still limited (AUC decreased to 0.85-0.90), and feature standardization and cross-device compatibility need to be optimized (49). Zhang et al. introduced a multi-instance learning framework, BBMIL/BBMIL-NA, to directly perform multiclass prediction of ER/PR/HER2, PAM50, five immune-related gene signatures, and two prognosis-related novel subtypes (TIP hot/cold phenotype and a cell-death subtype) from routine H&E WSIs across five datasets (2,599 WSIs). In TCGA-BRCA training/validation, AUCs for ER/PR/HER2/PAM50 were 0.883/0.788/0.703/0.803; across four external datasets they were 0.8982/0.7233/0.6412/0.6515. In CPTAC external validation, AUCs for immune signatures were 0.6362–0.7047; mean AUCs for TIP and cell-death subtypes were 0.752 and 0.694, respectively. An automatic tumor-ROI localization module achieved IoU 0.912, accuracy 95.22%, and AUC 0.993 on the BACH dataset, enabling a fully automated pipeline from WSI input to molecular/immune outputs (50).

## 4 Application of AI models in predicting lymph node metastasis in BC

BC lymph node metastasis is a core indicator for judging patients’ prognosis and formulating treatment plans. ALN is the most common metastatic site of BC, and its status directly affects the staging, surgical scope and adjuvant treatment decision (51). Although axillary lymph node dissection (ALND) can completely remove metastatic foci, it may cause complications such as upper limb edema, while SLNB, as a minimally invasive alternative, requires precise assessment of lymph node status to avoid overtreatment. However, axillary lymph node metastasis is present in about 30% to 40% of early-stage BC patients, and missed diagnosis of occult metastasis (e.g., micrometastasis) may affect survival, highlighting the critical importance of noninvasive assessment techniques (52, 53).

Ultrasound has become the first choice for axillary lymph node assessment due to its great advantages of non-invasiveness, real-time and low cost, but traditional diagnosis relies on subjective judgment and lacks standardized criteria. DL models significantly improve predictive efficacy by quantifying lymph node morphology, margins, and blood flow characteristics. Tahmasebi et al.’s AI system based on Google Cloud Auto ML Vision showed 74.0% sensitivity and 64.4% specificity in external validation (54). Although their study found that the sensitivity judged by high-level radiologists was at 89.9%, the higher specificity was indeed higher for the model, and the specificity judged by clinicians was only 50.1%. Sun et al. designed a 12-layer CNN model that achieved 65.5% sensitivity, 78.9% specificity, and an AUC as high as 0.72 for ultrasound image testing in 169 patients (55). In addition, the zhou and colleagues et al. et al. achieved 98% sensitivity, 99% specificity in 908 images by Kohonen self-organizing model. The AUC of their model was 0.97, which was significantly better than the AUC of 0.95 predicted by the feed-forward neural network model (56). Multimodal data integration further breaks through the limitations of unimodal data. Zheng et al. combined the DL features of conventional ultrasound with those of shear-wave elastography (SWE) and constructed a model to differentiate between metastatic states of lymph nodes, which predicted an AUC of 0.902, and the AUC of metastatic load, which predicted an AUC of Oh 0.905 (57). All of these predictions were significantly higher than the predictive performance of single modality. The model predicted an AUC of 0.719 for ultrasound and 0.759 for SWE in a single modality. Guo et al. simultaneously evaluated SLN and non-sentinel lymph node metastasis by the DL imaging histology model, and the sensitivity of SLN prediction reached 89.7% in 937 patients, with the model predicting an AUC of 0.81, and the sensitivity of non-SLN was 98.4%, with an AUC of 0.81 (58). The above model was the first to achieve the goal of distinguishing lymph node metastasis status. The above model was the first to achieve noninvasive assessment of non-SLN metastasis. Zhou et al. compared Inception and ResNet-101 architectures, of which Inception V3 was the best performer among 1,055 ultrasound images, with 85% sensitivity, 73% specificity, and 0.89 AUC, surpassing the average prediction level of 5 radiologists: 73% sensitivity, 73% specificity, and 0.89 AUC (59). The sensitivity of 85%, specificity of 73%, and AUC of 0.89 exceeded the average prediction of 5 radiologists: 73% sensitivity and 63% specificity. CT and MRI extend AI application scenarios through high resolution and functional imaging. the deformable attention VGG19 algorithm proposed by Liu et al. achieves 95% sensitivity and 86.75% specificity (AUC 0.9694) in contrast-enhanced CT (CECT), which outperforms the traditional ML models (e.g., RF, ResNet) (60). In MRI, the CNN model of Ren et al. CNN model had a sensitivity of 92.1% (AUC 0.91) in multicenter data, which was significantly higher than that of radiologists (AUC 0.80) (61). Yu et al. combined DCE-MRI imaging histology and clinical features to construct a nomogram, which predicted axillary metastasis with an AUC of 0.90 in 1,214 patients, which was better than imaging histology alone (AUC 0.85) or clinical model (AUC 0.71) (62).

## 5 AI-driven prediction of immunotherapy response and resistance mechanisms

### 5.1 AI in BC immunotherapy response prediction

AI-driven Imaging Histomics (Radiomics) technology has demonstrated excellent application value in the broader field of BC, especially in the prediction of treatment response and prognostic assessment. Triple-negative BC (TNBC) has been a difficult clinical diagnosis and treatment due to its high heterogeneity and complex prognostic factors (63, 64). In recent years, ML and DL technologies have revolutionized the prognostic prediction of TNBC. Li et al. innovatively constructed an MLIIC signature based on immune-infiltrating cell (IIC) characteristics from multi-omics data using 25 ML algorithms (65). The signature was optimized by identifying IIC-RNAs that were up-regulated in immune cells but down-regulated in TNBC cells and optimized by multiple algorithms, and was ultimately shown to significantly correlate with survival outcomes in TNBC patients and validated in multiple independent cohorts. Immunofluorescence staining further confirmed the prognostic value of the MLIIC signature, highlighting the critical role of the immune microenvironment in TNBC prognosis. Meanwhile, Albusayli et al. focused on the spatial features of the tumor microenvironment by applying DL algorithms to finely segment TNBC tissue images, quantify the spatial relationships between tumors, mesenchyme, and lymphocytes, and constructed digitized spatial tumor microenvironment (sTME) signatures, such as Digi-sTILs and Digi-TAS scores (66). The results showed that Digi-sTILs score and Digi-TAS score were strong prognostic indicators for disease-specific survival and were validated in a large cohort such as TCGA, with C-indexes of 0.65 and 0.60, respectively. This marks an important step in the field of AI-based digitized spatial features for precision medicine in TNBC. In addition, the study by Hou and colleagues addressed the highly heterogeneous nature of TNBC and delved into the relationship between tumor microenvironmental structures, especially the proportion of TLSs and tumor outgrowths (TBs), and clinical outcomes (67). They constructed a TLS/TB index by combining AI image analysis techniques to assess TLSs and TBs, and using multiplex immunofluorescence techniques to resolve cell subtypes within TLSs. It was found that the TLS/TB index was significantly and positively correlated with OS and RFS in TNBC patients, and that an elevated proportion of specific immune cell subpopulations (e.g., CD8+, CD45RO+, and CD20+ cells) within the TLSs also predicted a better survival prognosis (68). What’s more, the AI model they developed demonstrated performance beyond the traditional TNM staging system in predicting OS and RFS, highlighting the great potential of AI-driven structural analysis of the tumor microenvironment in TNBC prognosis prediction. In terms of prognostic prediction, imaging histology has been shown to be effective in predicting lymph node metastasis in BC. For example, imaging histology features based on DWI sequences are highly correlated with sentinel lymph node metastasis, which is expected to assist clinical decision-making and reduce unnecessary axillary lymph node dissection (69). In addition, imaging histology models can also predict Ki67 expression level, which is crucial for assessing tumor cell proliferative activity and guiding therapeutic choices, and studies have shown that imaging histology features based on both DCE-MRI and DBT are effective in predicting Ki67 expression, with AUC values of 0.773 and 0.698, respectively (70). What’s more, imaging histology has also achieved significant results in predicting the risk of survival and recurrence (71). The MRI-based imaging histology score was significantly correlated with disease-free survival and effectively predicted the recurrence score of multigene testing. For TNBC, DCE-MRI-based imaging histology features even capture peri-tumor heterogeneity as a prognostic factor for recurrence-free and overall survival (72). In the field of neoadjuvant therapy (NAT) response prediction, imaging histology also excels. Imaging histology models based on pre-treatment mpMRI are effective in predicting pathological complete remission (pCR) from neoadjuvant chemotherapy with AUC values up to 0.79, and combining it with molecular subtype analysis can further improve the prediction accuracy (73). PET/CT and ultrasound imaging histology, especially models combining deep-learning features, have also demonstrated the potential for predicting pCR and residual cancer burden (RCB), with AUC values up to 0.73 and 0.94, respectively (52, 74). Mao et al. proposed a fully automated multimodal pipeline, MIFAPS, which integrates pre-treatment MRI, pathology WSI, and clinical variables to predict pCR after NAC in a multicenter, prospective cohort (n = 1004). The model achieved AUC = 0.882 on the pooled external test sets and AUC = 0.909 on the prospective test set, significantly outperforming any single-modality model (P<0.05). Biological analyses showed that high-score cases were enriched for immune-related pathways and associated with upregulation of memory CD4 T cells and M1 macrophages, indicating concordance between imaging signals and an active immune microenvironment. The authors further illustrated treatment-allocation scenarios based on model-driven stratification (75). Huang et al. enrolled 2,279 patients from 12 centers receiving NAT and built a longitudinal, multimodal model combining MRI “habitat” radiomics, bulk transcriptomics, and scRNA-seq to predict pCR and delineate the immune landscape. The model yielded AUCs of 0.863, 0.813, and 0.888 in the external validation, immunotherapy, and multi-omics cohorts, respectively, remaining robust across molecular subtypes and clinical stages. Patients with higher model scores exhibited significantly greater B-cell infiltration (B-cell score, p = 0.00039), supporting a biological link between longitudinal imaging phenotypes and the TIME (76).

### 5.2 AI for predicting drug resistance in BC

**Figure 2:**
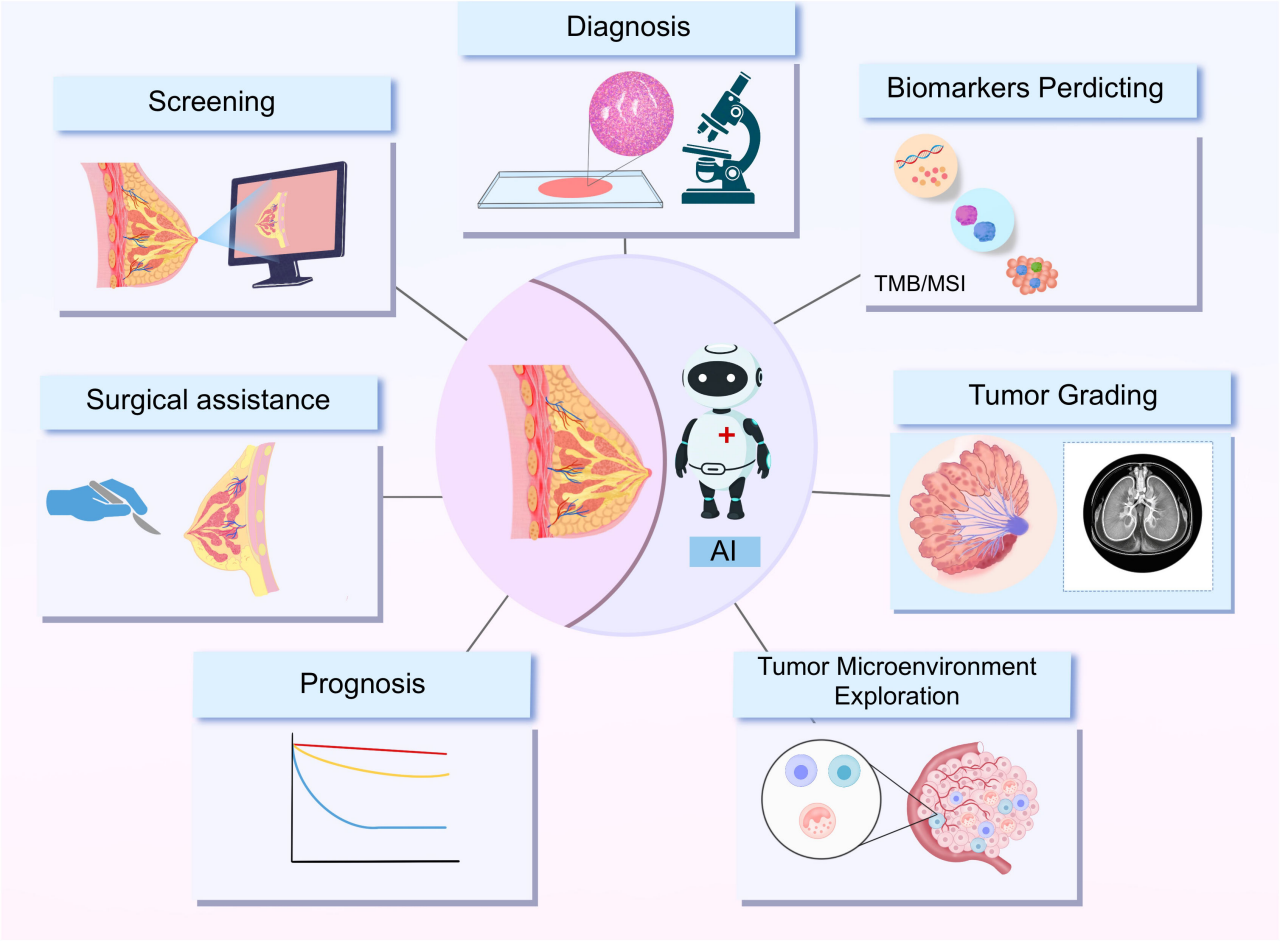
AI based radiomics data in breast cancer.

The research application of artificial intelligence technology in tumor drug resistance is bringing a great new breakthrough in the field of cancer treatment. Tumor drug resistance, as the key to the failure of chemotherapy, targeted therapy and immunotherapy, is mainly affected by the multi-dimensional interactions of the tumor microenvironment, the highly heterogeneous nature of tumor cells, and the dynamic regulation of epigenetics at multiple levels (77). Traditional histological studies are difficult to systematically analyze its dynamic changes, and artificial intelligence technology, with its powerful data processing and pattern recognition capabilities, has shown great advantages in analyzing the molecular mechanism of tumor cells at the level of molecular mechanism, drug development, and the prediction of treatment plans. A multi-omics analysis based on molecular characterization and adriamycin treatment response in BC patients showed that only 1.56% (2/128) of the models demonstrated significant predictive efficacy in a systematic test of 16 machine learning algorithms with 8 types of molecular profiles (78). The optimal model was a four-variable nonlinear classifier constructed based on the CART algorithm, which, by integrating the expression profiles of four miRNAs, namely, hsa-miR-21-5p, hsa-miR-155-5p, hsa-miR-34a-3p, and hsa-miR-200c-3p, achieved a median MCC = 0.56 for the prediction of azithromycin resistance and an AUC of 0.80 with excellent results. The model performance curve gradually increased with the increase of training sample size, and the AUC could be improved by 0.07 for every 100 cases of sample increase (p<0.001). Nada et al. developed a DL method to cluster 9068 small molecule inhibitor libraries by employing a gradient boosting decision tree with molecular fingerprinting features, and found that the N-substituted quinazolin-4-amine analogs had the highest percentage of compounds, about as 27.6% (79). On the basis of this structure, a novel 4-anilinoquinazoline derivative was designed by CADD strategy, which was predicted by DL model with 89.2% accuracy. Immediately followed by the synthesis of 18 highly active candidate molecules, it was found that compound 9 ((E)-N-(3-chloro-4-fluorophenyl)-6-(4-(dimethylamino), but-2-enamido) quinazolin-4-amine) exhibited significant antitumor activity. The IC50s against MCF-7 cells were 2.50 ± 0.21 μM and 1.96 ± 0.18 μM, respectively, which were 1.8-fold elevated compared with the positive control gefitinib. The IC50 for EGFR kinase inhibition amounted to 2.53 ± 0.15 nM, while the apoptosis-inducing rate was elevated to 64.3%. The study reduced the conventional drug development cycle by 37% through a strategy combining virtual screening and synthetic validation, providing a new chemical entity for overcoming EGFR inhibitor resistance. A postoperative prognostic predictive modeling study of TNBC based on programmed cell death (PCD) patterns integrates 12 PCD mechanisms and constructs 12-gene marker cell death indices (CDIs) from seven multi-omics cohorts, including TCGA (n=1,132) and METABRIC (n=1,904), via the Stacked-Cox algorithm (80). The study showed that the 5-year postoperative overall survival rate was significantly lower in the high CDI group than in the low CDI group (p<1e-6). The concordance index C-index reached 0.73-0.81 in five independent datasets. single-cell transcriptome analysis revealed a 2.8-fold increase in the proportion of Treg cells in the high CDI tumor microenvironment and up-regulation of the expression of immune checkpoints such as PD-L1 and CTLA4. Drug sensitivity prediction showed that patients with high CDI were resistant to docetaxel (OR = 0.32, p=0.008), oxaliplatin (OR = 0.29, p=0.003), but sensitive to pabocinib (64.7% reduction in IC50, p=0.001). AI has moved beyond single−marker associations to integrative predictors that anticipate resistance before therapy starts and guide alternative regimens. In ER−positive disease, an annotation−free H&E−based framework accurately predicted epithelial–mesenchymal transition (EMT) phenotype and endocrine response (overall accuracy 81.25%), with 88.7% of endocrine−resistant slides classified as mesenchymal and 75.6% of sensitive slides as epithelial, linking routine morphology to resistance−relevant biology (81). Pre−treatment genomic, transcriptomic, and immune−ecosystem features were integrated by machine learning to show a monotonic relationship between residual disease and baseline tumor ecology, indicating that resistance risk is encoded in the pre−therapy landscape and is predictable. For cytotoxic regimens, interpretable multi−feature models predicted paclitaxel response, while PET/MRI and MRI radiomics/deep learning markedly improved pCR prediction to neoadjuvant chemotherapy, enabling early identification of non−responders for treatment adaptation (82). Pharmacogenomic approaches further support personalization: ML models highlight the role of CYP2D6 metabolizer status in tamoxifen benefit, and resistance−gene risk signatures stratify outcomes in HER2−positive breast cancer (83). To generalize across drugs and tumor contexts, modern methods couple drug molecular graphs with tumor gene−expression profiles and learn predictive mappings on large pharmacogenomic resources (GDSC/CCLE). Recent models (such as explainable graph−based architectures and DIPK) report improved IC50 prediction and transfer performance, providing technical foundations for breast−specific models. Few−shot graph neural networks that embed prior biological knowledge further enhance accuracy when sample sizes are limited, a common constraint in subtype−specific resistance studies (84). At the therapeutic−design end, AI accelerates hit identification and lead optimization: explainable models have prioritized new candidate compounds; one pipeline achieved 89.2% predictive accuracy and produced a quinazoline derivative with MCF−7 IC50 ≈2 μM and EGFR IC50 2.53 nM, shortening the discovery cycle by 37% (85, 86). In parallel, generative AI and LLM−guided frameworks are transforming antibody design, enabling rapid sequence exploration and affinity maturation. Here, we summarize the application of AI to breast cancer based on radiomics data (**Figure 2**).

Pipeline steps: Segmentation of the lesion (e.g., on mammography) → Feature extraction (shape, first−order/histogram, texture, and transform−domain features) → Data analysis (quality control, feature stability, correlation pruning) → Model building (classification, regression, or survival models with calibration) → Precision medicine outputs, including virtual biopsy, benign–malignant diagnosis, molecular subtype surrogates, axillary status, prediction of pCR/therapy response, and prognosis. Outputs are intended to be explainable and linked to actions such as recall, biopsy, surgery planning, systemic therapy, and follow−up.

## 6 Current challenges and future directions in the application of AI in radiomics of BC

### 6.1 Current challenges

AI in BC research, despite showing amazing potential, has multiple challenges to overcome to mature its application in clinical practice. The limitation of model validation is the primary challenge. The validation process of imaging genomics models established in many current studies often relies on single-center, retrospective datasets, which severely limits the models’ ability to generalize and their reliability across different healthcare institutions. More critically, existing models lack validation in prospective clinical trials, making it difficult to assess their performance in real clinical scenarios and their superiority compared to existing gold standard therapies. Therefore, large-scale, multicenter prospective studies are urgently needed to establish the clinical value of AI models in the real world. In addition, the lack of interpretability of models is an important factor hindering the clinical application of AI (87, 88). DL imaging genomics models, especially end-to-end models, have high predictive accuracy, but their intrinsic “black box” nature makes it difficult for clinicians to understand the decision-making basis of the models, thus reducing trust and willingness to apply them. Even for traditional imaging genomics models, the correlation mechanism between the extracted manual features and the biological events of tumors is still unclear, limiting the explanatory power of the model at the biological level. How to improve the interpretability of AI models and build more transparent and easier to understand models is an important direction for future research. Finally, multimodal data integration and system integration also face challenges (89, 90). The future development trend is to integrate multi-dimensional data such as genomics, imaging genomics, pathomics, etc., to construct more comprehensive radio-multi-omics models, but this needs to overcome the technical difficulties of integrating and analyzing heterogeneous data from multiple sources (91). Meanwhile, in order to translate the potential of multiple clinical applications of AI models into reality, it is necessary to develop a user-friendly, integrated AI-assisted system that integrates multiple functions, such as tumor classification, prognosis prediction, and treatment response assessment, and provides personalized, interpretable reports to enhance the system’s utility and acceptability in the clinical environment.

### 6.2 Future directions

To address the above challenges, the future development of AI in BC should focus on the following key directions. First, it is crucial to optimize model validation strategies. Future studies should place more emphasis on multicenter validation and actively utilize large-scale, multicenter cohorts or open databases for external validation in order to enhance the generalizability and clinical applicability of models. Meanwhile, prospective clinical trials should be actively promoted to push models that perform well in retrospective studies to a higher level of evidence, comprehensively assess their clinical value, and compare them with existing treatment regimens to provide more solid evidence for clinical application (92, 93). Second, improving model interpretability is the key to winning clinical trust. Future research should explore the interpretability of DL radiomics, for example, to parse the decision-making process of DL models by technical means, or to extract DL features and then combine them with traditional ML methods for modeling, in order to ensure the prediction accuracy while enhancing the interpretability of the models. Further, the research on the association between imaging genomics and biological events should be deepened, evolving from radio-genomics to multi-omics, integrating multi-omics data, and digging deeper into the intrinsic connection between imaging features and tumor biological behaviors, so as to enhance the biological explanatory power of the model, and thus better guide the clinical practice (94, 95). Finally, the construction of an integrated AI-assisted system is a must for realizing the clinical translation of AI. Future research should learn from the successful experience of CAD systems in BC screening, develop a multifunctional integrated system that integrates multiple functional modules from tumor classification to prognosis prediction and treatment response assessment, and focus on improving the user-friendliness of the system, lowering the threshold of use for both doctors and patients through simple and intuitive user interfaces and detailed and interpretable reports, and ultimately realizing the seamless integration of AI technology in the diagnosis and treatment process of BC (96, 97). Ultimately, the system will realize the seamless integration of AI technology in the diagnosis and treatment process of BC, and comprehensively improve the diagnosis and treatment level of BC and the survival benefit of patients.

## 7 Conclusion

The clinical challenges of breast cancer concentrate on two fronts. A considerable proportion of patients present at an advanced stage at first diagnosis, and pronounced molecular and immune heterogeneity leads to substantial variability in treatment response. Recent evidence has progressed from proof of concept to real-world and prospective evaluations. In population screening, AI-assisted reading can maintain or even increase cancer detection while typically reducing the radiologists’ reading workload by about half, providing a scalable pathway to achieve earlier detection and intervention under workforce constraints. These findings indicate that AI is not only a means to improve isolated model metrics but also a system-level enabler that enhances screening efficiency and overall care quality. Looking ahead, two directions are crucial. First, multimodal and longitudinal integration: coupling mammography, ultrasound, and MRI with digital pathology, bulk transcriptomics, and single-cell omics can noninvasively quantify the tumor immune microenvironment and improve stratification for pCR, immunotherapy response, and drug-resistance risk, thereby supplying actionable probabilistic evidence for individualized treatment. Second, clinical translatability and governance: large-scale, multicenter, temporally external prospective validation is required, with routine reporting of endpoints directly linked to clinical action, including calibration, decision-curve net benefit, stage shift, PPV, recall, and workload, rather than discrimination alone. As these methodological and governance elements mature, AI can be embedded into clinical workflows in an explainable, governable, and affordable manner, enabling end-to-end precision from early detection to treatment-response prediction and ultimately improving patient survival and quality of life.
